# Pancreatic cancer stem cells in patient pancreatic xenografts are sensitive to drozitumab, an agonistic antibody against DR5

**DOI:** 10.1186/s40425-016-0136-y

**Published:** 2016-06-21

**Authors:** Jason W.-L. Eng, Thomas A. Mace, Rohit Sharma, Danielle Y. F. Twum, Peng Peng, John F. Gibbs, Rosemarie Pitoniak, Chelsey B. Reed, Scott I. Abrams, Elizabeth A. Repasky, Bonnie L. Hylander

**Affiliations:** Department of Immunology, Roswell Park Cancer Institute, Buffalo, NY 14263 USA; Present Address: Division of Medical Oncology, Department Internal Medicine, The Ohio State University, Columbus, OH 43210 USA; Department of Surgical Oncology, Roswell Park Cancer Institute, Buffalo, 14263 NY USA; Present Address: Department of Surgery, Lehigh Valley Physician Group, Allentown, 18103 PA USA; Present address: Department of Surgery Chief of Surgical Oncology, Jersey Shore University Medical Center, 1945 State Highway 33, Neptune, NJ 07753 USA

**Keywords:** Pancreatic cancer, Stem cells, Apo2L/TRAIL, Drozitumab, Death receptor

## Abstract

**Background:**

Therapeutic resistance and tumor recurrence are two major hurdles in the treatment of pancreatic ductal adenocarcinoma. Recent findings suggest that both of these attributes are associated with a small subset of pancreatic tumor initiating cancer stem cells (CSCs). Here, we demonstrate that drozitumab, a human agonistic monoclonal antibody which binds the death receptor DR5, selectively eliminates CSCs, resulting in tumor growth inhibition and even regression of pancreatic tumors.

**Methods:**

To examine the efficacy of drozitumab against pancreatic CSCs, we treated patient-derived pancreatic tumor xenografts (PDX) in immunocompromised SCID mice and evaluated tumor control. To assess apoptosis following drozitumab treatment, we identified the CSCs as CD24+, CD44+, and EpCAM+ by FACS analysis, and measured in vivo and in vitro levels of cleaved caspase-3. Lastly, in vitro evaluation of DR5 re-expression was performed using isolated patient pancreatic cancer xenograft cells along with the cell line, Panc-1. After treatment with drozitumab, the remaining DR5- cells were assessed by FACS analysis for DR5 expression at the cell surface at 8, 24 and 48 h post-treatment. All in vivo growth data was analyzed by 2-way Anova, incidence data was analyzed using Mantel-Cox, and in vitro studies statistics were performed with a t-test.

**Results:**

We find that while 75–100 % of CSCs express DR5, only 25 % of bulk tumor cells express the death receptors at any one time. Consequently, drozitumab treatment of SCID mice bearing PDX kills higher percentages of CSCs than bulk tumor cells. Additionally, SCID mice implanted with isolated CSCs and then immediately treated with drozitumab fail to ever develop tumors. In vitro studies demonstrate that while drozitumab treatment reduces the DR5+ cell population, the remaining tumor cells begin to express DR5, suggesting a mechanism by which continuous administration of drozitumab can ultimately result in tumor regression despite the initially low percentage of DR5+ cells.

**Conclusions:**

Overall, our work reveals that treatment of pancreatic tumors with the drozitumab can lead to long-term tumor control by targeting both bulk cells and CSCs.

**Electronic supplementary material:**

The online version of this article (doi:10.1186/s40425-016-0136-y) contains supplementary material, which is available to authorized users.

## Background

Pancreatic ductal adenocarcinoma remains one of the most difficult forms of solid cancer to treat and, as the population ages, is projected to become the second leading cause of cancer related deaths by 2030 [1]. The current first-line therapy for patients is the nucleoside analogue, gemcitabine; however, this therapy only offers palliative care. Several other cytotoxic and targeted therapies have been tried in combination with gemcitabine, but these also have not improved overall survival with the exceptions of erlotinib [2], FOLFIRINOX, which has limited usage in patients due to toxicity [3] and *nab-*paclitaxel [4]. It has been suggested that one reason for the therapeutic resistance of pancreatic (and other) cancers could be resistance of resident cancer stem cells to standard therapies and that therapies which target CSCs could potentially improve survival [5].

Pancreatic CSCs have been identified both in patient tumors by their expression of the cell surface markers ESA (epithelial surface antigen; CD326), CD44 and CD24 [6] and in murine pancreatic tumors with a slightly different panel of markers (CD44+, CD133+, Sca1+) [7]. While CSCs comprise an extremely small proportion of the total tumor mass, several studies have revealed their high tumor-forming potential (e.g. [6, 8, 9])*.* Additionally, these cells possess an increased level of resistance against many standard therapies [3]. In patient pancreatic tumor xenografts, Simeone et al. found that CSCs survive and become enriched following radiation or gemcitabine treatment [5]. Since CSCs persist after treatments which kill bulk tumor cells in several types of tumors [10–12], these cells are implicated in the regrowth of tumors in patients and have become a major focus as a therapeutic target [13].

In previous work, we showed that Apo2L/TRAIL, a recombinant form of TRAIL, a tumor necrosis factor (TNF) family member which binds to the cell surface death receptors DR4 and DR5 and initiates apoptosis through the extrinsic apoptotic pathway, can effectively inhibit tumor growth in several PDX models of pancreatic cancer [14, 15]. Binding of Apo2L/TRAIL to its receptors results in the activation of the extrinsic apoptotic pathway leading to cell death. Unlike other members of the TNF family, Apo2L/TRAIL has minimal effects on normal healthy tissues, making it a promising therapeutic agent for treating cancer [16]. However, Apo2L/TRAIL has a relatively short lifespan of approximately 30 min in circulation due to its rapid degradation and clearance [17]. Therefore, humanized or human agonistic monoclonal antibodies (which have a half-life from several days to weeks) have also been developed to target either DR4 or DR5 [16, 18–20].

In this study, we found that the anti-DR5 antibody, drozitumab (see [19] for details of this antibody), used alone, inhibits the growth of pancreatic cancer patient xenografts. Based on these promising responses, we questioned whether CSCs were sensitive to drozitumab. In both in vitro and in vivo experiments, examination of the levels of apoptosis in CSCs immediately following treatment indicates that CSCs in these tumors are extremely sensitive to drozitumab. Furthermore, our data shows that while almost all the CSCs express DR5, DR5 is expressed by only a fraction of bulk tumor cells. To determine how the bulk tumor responds to drozitumab when only a fraction of the cells expressed DR5, we investigated death receptor expression kinetics in vitro using both a commercial pancreatic cancer cell line and cells isolated from a PDX. These results demonstrate that cell surface DR5 expression is dynamic, and following killing of DR5+ cells, a portion of the DR5- cells express DR5. Altogether, our results indicate that pancreatic CSCs are sensitive to treatment with drozitumab and provide further rationale for exploring the use of anti-DR5 agents with current therapeutic regimens to improve tumor control.

## Results

### Patient derived pancreatic xenograft tumors are sensitive to drozitumab

**Fig. 1:**
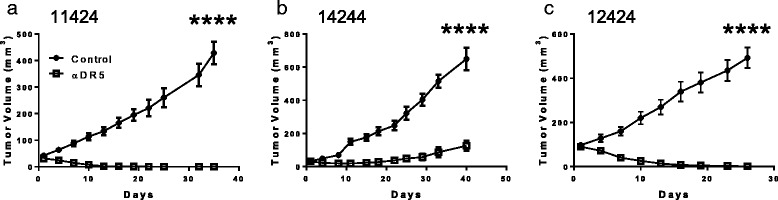
The growth of three different established patient-derived xenografts is inhibited by treatment with drozitumab. SCID mice implanted with tumors **a**) 11424 and **b**) 14244 were given weekly intraperitoneal injections of drozitumab. **c**) Mice with xenograft 12424 were given drozitumab daily by intraperitoneal injection. *n* = 5–9 mice/group. Statistics by 2-way Anova and Bonferroni post-test; ****p <0.0001

To evaluate their sensitivity to drozitumab, patient tumor xenografts previously identified as sensitive (11424 and 14244) or resistant (12424) to Apo2L/TRAIL were implanted into immunodeficient SCID mice and treated in vivo. Xenografts 11424 and 14244 showed a significant response to drozitumab when the antibody was administered weekly (Fig. 1a and b) and complete regression of 11424 was seen within four weeks. Interestingly, tumor 12424 did not respond when mice were treated with drozitumab 1× or 3×/week (Additional file 1: Figure S1); however, when the mice were treated daily, the tumor regressed (Fig. 1c), suggesting that increasing the circulating levels of the antibody could overcome the apparent resistance of certain tumors to drozitumab.

### Pancreatic CSCs are sensitive in vivo to treatment with drozitumab

Work from several groups suggests that CSCs comprise a small population within the tumor microenvironment, but are significantly more resistant to therapies than the terminally differentiated, bulk tumor cells [21, 22]. Therefore, we investigated whether the pancreatic CSCs were also resistant to drozitumab. We first purified CSCs by FACSort using the cancer stem cell markers ESA^+^CD24^+^CD44^+^ [6]; a marker for the murine MHC class I molecule, H-2K^d^, was also included to enable identification and exclusion of murine stromal cells from the analysis. To confirm that these putative CSCs possessed enhanced tumorigenic properties, we implanted SCID mice with different numbers of the cells and monitored for tumor growth. Compared with bulk tumor cells, the purified CSC populations were able to generate tumors when as few as 10^4^ cells were injected. Histological examination of the tumors derived from the cancer stem cells revealed that the morphology and architecture resembled that of the original parent xenograft, indicating that the purified CSCs alone could generate tumors (Additional file 1: Figure S2).

**Fig. 2:**
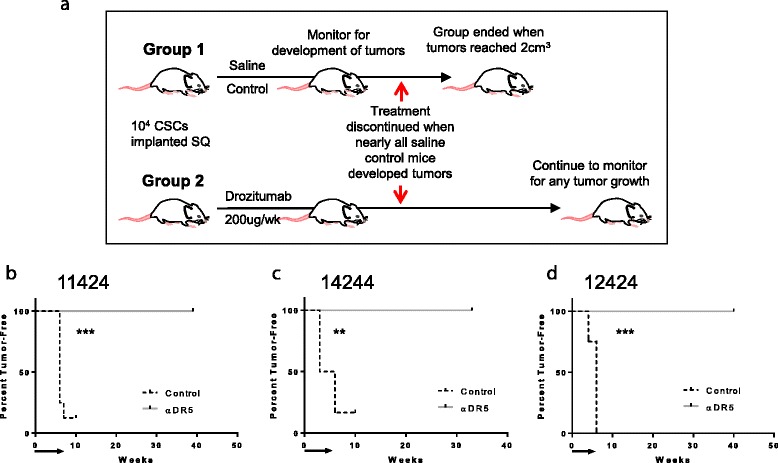
Treatment of CSCs in vivo with drozitumab (α-DR5) prevents tumor development. **a**) Experimental design schematic. Purified triple positive cancer stem cells from **b**) 11424, **c**) 14244, and **d**) 12424 were engrafted in SCID mice and mice were immediately treated with either vehicle (control) or drozitumab until the majority of control mice developed tumors. *Arrows* indicate duration of treatment. After treatment with drozitumab was discontinued, tumor-free mice were monitored for tumor development. *n* = 6–8 mice/group. Statistics by Mantel-Cox test; **p <0.01, ***p <0.001

To characterize the response of pancreatic CSCs to anti-DR5 targeted therapy in vivo, 10^4^ ESA^+^CD24^+^CD44^+^ cells were injected SQ into SCID mice and the mice were then treated weekly with either vehicle or drozitumab. Treatment was continued until nearly all mice receiving the vehicle control developed palpable tumors (Fig. 2a). At that time, treatment with drozitumab was discontinued and the treated mice were monitored for the development and growth of tumors. We hypothesized, based on the previous inhibition of tumor growth by drozitumab, that measureable tumors would not develop during the treatment period, but that if CSCs were resistant to drozitumab, they would survive and palpable tumors would subsequently develop once drozitumab administration ceased. Surprisingly, mice which received drozitumab failed to develop tumors even several months after treatment ended (Fig. 2b-d), suggesting that drozitumab treatment completely eliminated cells capable of initiating tumor growth.

### Pancreatic CSCs express high levels of death receptors

**Fig. 3:**
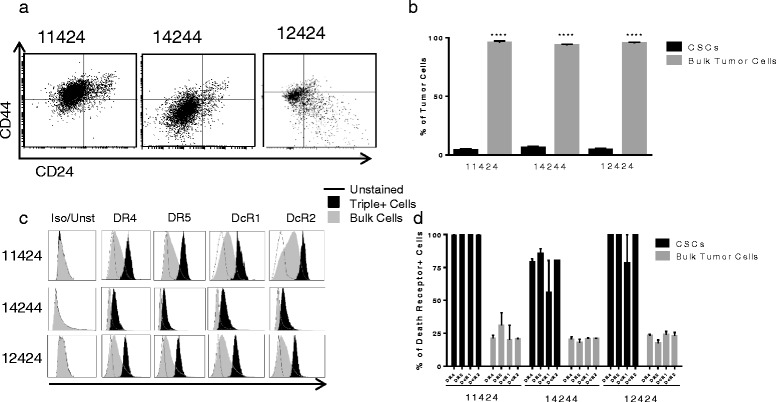
CSCs express higher levels of death receptors compared with bulk tumor cells. **a** Gating strategy for identifying cancer stem cells. Tumors were dissociated with type IV collagenase and gated on ESA+ cells (not shown), followed by CD24+ and CD44+ populations. **b)** Percentage of cancer stem cells in patient xenografts 11424, 14244 and 12424. **c**) Representative plots of DR4, DR5, DcR1, DcR2 expression on triple positive cancer stem cells and bulk tumor cells compared with unstained controls. Background staining of isotype controls (Iso) was comparable to unstained cells (Unst). **d**) Analysis of percentage of cells expressing DR4, DR5, DcR1 and DcR2. Statistics by Student t-test; ****p <0.0001. Experiment performed three times in triplicate

To elucidate the mechanism by which drozitumab treatment abrogated tumor development in vivo, death receptor expression on the surface of tumor cells was quantified by flow cytometry. As previously shown [6], CSCs comprise a small population of cells within the tumor (Fig. 3a, b). We found that the expression of death receptors, DR4 and DR5, as well as the decoy receptors, DcR1 and DcR2, was significantly higher in the CSC population compared with the bulk tumor cells (approximately 85–100 % of CSCs expressed death receptors compared with only 25 % of bulk cells; Fig. 3a). These data correlate with our observations that pancreatic CSCs are extremely sensitive to drozitumab treatment.

### CSCs are preferentially sensitive to in vitro killing by drozitumab

**Fig. 4:**
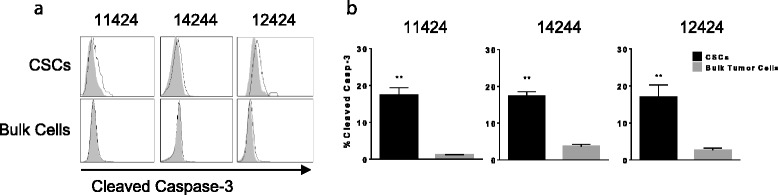
Cancer stems cells undergo apoptosis following treatment with drozitumab. **a**) Dissociated tumor cells from 11424, 14244 and 12424 treated in vitro with 10 μg/ml of drozitumab antibody for 1 h and incubated for 7 h with 10 μg/ml of anti-human Fc IgG antibody. Cells were stained for ESA+, CD44+, CD24+ and cleaved caspase-3 and analyzed by flow cytometry. **b**) Percent of CSCs and bulk tumor cells with cleaved caspase-3. Statistics by Student t-test; **p <0.01. Experiment performed three times

First, to determine if higher percentages of the CSCs than bulk cells were killed by anti-DR5 antibody, PDXs were manually dissociated and then treated in vitro*.* Cultured cells were treated initially for 1 h with 10 μg/mL of drozitumab followed by 7 h with a crosslinking anti-human Fc IgG antibody [23, 24]. Bulk tumor cells and CSCs were then analyzed for the levels of cleaved caspase-3 by flow cytometry. Results of these experiments revealed that drozitumab treatment resulted in higher percentages of apoptosis of CSCs than bulk tumor cells (Fig. 4).

### Treatment of patient xenograft tumor-bearing mice with drozitumab preferentially induces apoptosis of CSCs

**Fig. 5:**
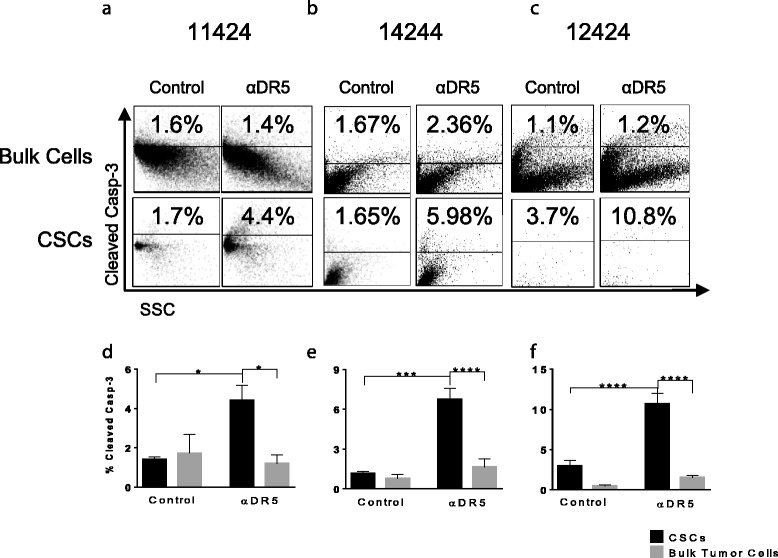
Treatment of tumor bearing mice in vivo with drozitumab induces apoptosis primarily in triple positive cells. Mice were treated with 200 μg drozitumab and tumors were resected, disaggregated and percentage of cleaved caspase-3 in CSCs vs. bulk tumor cells analyzed by flow cytometry. Representative flow plots and quantification of cleaved caspase-3 in patient xenografts: **a** & **d**) 11424, **b** & **e**) 14244 and **c** & **f**) 12424. *n* = 3 mice/group. Statistics by Student t-test; *p <0.05, **p <0.01

To determine whether the higher proportion of CSC killing seen in vitro also occurred in vivo, we treated mice bearing patient tumor xenografts with a single dose of drozitumab. After 5 h, tumors were collected and analyzed for caspase-3 activation. Similar to the in vitro studies (Fig. 4), cleaved caspase-3 was observed in a significantly higher percentage of the CSCs compared with the bulk tumor cells or CSCs from vehicle-treated controls (Fig. 5).

### Following depletion of DR5+ cells, DR5- cells begin to express DR5

**Fig. 6:**
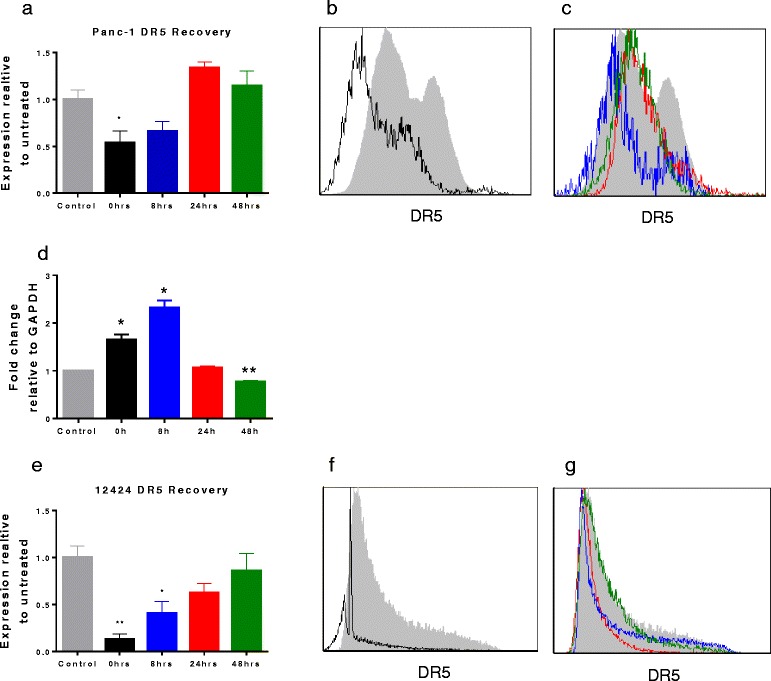
Drozitumab treatment depletes DR5+ cells in vitro. Subsequently, DR5 is increasingly detected on residual cells and reaches pre-treatment levels by 48 h post-treatment in both Panc-1 (a human cell line, **a-d**) and cells derived from a patient xenograft (12424, **e-g**). Cells were treated in culture for 10 h with 10 μg of drozitumab and 10 μg of α-human IgG Fc antibody and cells were assessed for DR5 expression immediately after treatment (0 h) or intermittently at 8, 24 and 48 h following treatment. **a, e**) Plots of expression relative to untreated controls. **b**, **c** Representative flow plots from Panc-1. **d** Fold change of DR5 mRNA in Panc1 cells compared to untreated controls. **f, g** Representative flow plots from 12424 PDX cells. Statistics by Student t-test; *p <0.05, **p <0.01 compared to untreated controls. Experiment performed twice in triplicate. (Colors in **b, c** and **f, g** represent times post-treatment illustrated by bars in **a** and **e** respectively)

As only approximately 25 % of the bulk tumor cells are DR5+ at the time of analysis, we questioned how continued treatment with drozitumab leads to tumor growth inhibition and regression seen in Fig. 1. We hypothesized that following drozitumab induction of apoptosis in the DR5+ cells, the residual DR5- cells must subsequently express cell surface DR5. To determine if the overall response of the tumor to drozitumab was due to upregulation of expression of DR5 on DR5- cells, we quantified the number of DR5+ tumor cells in vitro following treatment with the antibody. The human pancreatic cancer cell line Panc-1 and cells isolated from the patient xenograft 12424, were treated with drozitumab in serum-free media for 10 h to ensure maximal killing of DR5+ cells and then stained for flow cytometry using drozitumab to detect DR5. Immediately following treatment (0 h), there was negligible expression of DR5 on the surface of the surviving tumor cells (Fig. 6a, b and e, f). This was reflected in a substantial decrease in the expression of DR5 mRNA in the remaining cells following drozitumab. mRNA expression peaked rapidly at 8 h post-treatment and then subsequently declined by 48 h (Fig. 6d). Additionally, treatment with drozitumab led to significant depletion of CSCs in both the Panc-1 cell line and the dissociated 12424 cells (Additional file 1: Figure S3A and B). Surviving DR5- cells were washed and allowed to recover; after 8 h of recovery, low level DR5 expression was detectable on the surface of these cells (Fig. 6a, c and e, g) and by 48 h, the expression of DR5 on the cell surface was comparable to the levels of the untreated control cells (Fig. 6a, c and e, g). These findings support the notion that DR5 expression on formerly DR5- tumor cells permits continued drozitumab-mediated killing of sensitive tumors even when only ~25 % of the malignant cells express this death receptor at any given time. In summary, these data demonstrate that drozitumab can control pancreatic tumors by potentially targeting both CSCs and DR5 expressing bulk tumor cells.

## Discussion

Cancer stem cells present a unique challenge to more effective treatment outcomes since these cells survive traditional therapies such as chemotherapy and radiation therapy [25, 26] that kill the majority of the bulk tumor cells. Therefore, CSCs have the potential to facilitate recurrence and significantly impact patient outcome in spite of their small numbers. For this reason, it is important to develop novel approaches for targeting them.

Our current data demonstrates that nearly all pancreatic CSCs express cell surface DR5, and provides encouraging evidence for the effectiveness of DR5-directed treatment against this cell population. These findings corroborate previous studies in pancreatic [27] and breast cancer [28] that revealed that a different anti-DR5 antibody, TRA8 (tigatuzimab), also has efficacy against CSCs. However, our work demonstrates for the first time that a different, independently developed antibody, drozitumab, which may recognize a different epitope, has potent anti-pancreatic CSC efficacy. Furthermore, we performed these experiments using pancreatic PDX tumors with previously characterized responses to Apo2L/TRAIL treatment, but not to anti-DR5 antibody therapy. Interestingly, we found that the CSCs from both Apo2L/TRAIL sensitive and resistant tumors also express elevated levels of DR4 and both decoy receptors, DCR1 and DCR2. Yet, despite the presence of these decoy receptors, drozitumab still induced apoptosis in the CSCs. As previously reported, drozitumab has high specificity for DR5, which may contribute to its efficacy against the CSCs in spite of the high numbers of decoy receptors [19]. Our findings indicate that targeting DR5 alone is sufficient to eliminate the CSC population, and that drozitumab may be an effective therapy even against tumors which are resistant to other members of the TNF family, including Apo2L/TRAIL.

Previous studies of pancreatic CSCs used the markers Aldefluor+CD24+CD44+ to identify a slightly different population of cells [27]. As noted in Penchev et al. these heterogeneous populations could behave differently and represent distinct tumor initiating cell populations [29]. In fact, several studies have demonstrated variability in the expression of death receptors across these populations. For instance, Fu et al. found that CSCs labeled with CD133+, ESA+, CD24+, CD44+ isolated from primary pancreatic tumor specimens expressed negligible levels of DR4 and DR5; however DR4 and DR5 were upregulated by inhibition of hedgehog signaling and, in humanized SCID mice, this led to slowing of tumor growth [30]. On the other hand, a study on several colon cancer cell lines found that DR4 was more highly expressed on the CSCs identified as a side population with FACS analysis. These cells were actually more sensitive to TRAIL induced apoptosis, even though DR5 expression was similar between the side population cells and the bulk tumor cells [31]. In other tumors, the CSCs were found to be resistant to death receptor targeting. For example, the detached spheroids, a widely accepted technique used to isolate CSCs, were resistant to TRAIL treatment due to a lack of expression of DR4 (BT20) or DR4/DR5 (MCF-7) expression [32] compared to the adherent cell populations. Recent studies in medulloblastoma and glioma found similar responses [33]; however, in the case of glioma, the non-stem cell population displayed moderate sensitivity to TRAIL, as opposed to the stem cells which were resistant partly due to a lack of caspase-8 [34]. Overall, the sensitivity of CSCs to death receptor activation seems to vary depending on specific tumor type and further studies are required to assess the response of different CSC populations to death receptor activation.

Although the majority of bulk tumor cells do not express detectable levels of DR5, the continued regression of established tumors by drozitumab treatment (Fig. 1) suggests that these cells may upregulate the receptor. Our analysis of these cells in vitro indicates that DR5 receptors continuously appear on the tumor cells, allowing for sustained antibody efficacy. Mechanistically, tumor regression appears to be mediated both by elimination of the CSC pool, leading to an inability to repopulate the tumors, and also by targeting of the bulk tumor cells. Interestingly, our data also reveals that nearly all of the CSCs in the patient tumors express DR5 and DR4; however, only approximately 20 % of CSCs undergo apoptosis at any given time-point. We suspect that this discrepancy between the receptor expression and the level of cell death results from individual variations in DR expression even among CSCs. In addition, the data reflects the maximum peak of apoptosis, but cell death also begins prior to this time point and residual cells may continue to undergo apoptosis afterwards. Thus, a substantial population of cells ultimately undergoes apoptosis after treatment with drozitumab. The fact that slightly lower levels of apoptosis are detected in tumors following in vivo exposure suggests that the kinetics of delivery and/or exposure may differ in vivo.

Moreover, our in vitro findings with the Panc-1 cell line surprisingly suggest that the re-emergence of the receptor depends on *de novo* synthesis in the remaining cells. Intriguingly, the expression of DR5 mRNA peaks at 8 h post-treatment, indicating that a form of steady state has been reached. Further investigation will be necessary in the future to elucidate the mechanisms regulating this process.

It is also encouraging that the CSCs in a relatively Apo2L/TRAIL resistant tumor (12424) appear to respond to drozitumab just as well as the Apo2L/TRAIL sensitive tumors. Recently, Chaffer et al. demonstrated that in mammary tumor cell lines, non-stem cells could give rise to stem cells both in vitro and in vivo [35]. In the future, it will be important to determine whether the increase in DR5 expressing cells we observed 48 h after depletion represents new expression of DR5 by previously DR5- bulk tumor cells or the conversion of DR5- bulk tumor cells to DR5+ CSCs.

Despite promising pre-clinical data, several clinical trials testing drozitumab and other DR5 targeting therapies have shown mixed results [18]. In certain instances, these trials have revealed that anti-DR5 mAb treatments could induce stable disease in patients with GI malignancies and reduced tumor burden in cases of granulosa ovarian cancer and chondrosarcoma, but not in other tumor types [36, 37]. Although this lack of clinical efficacy of both anti-DR5 antibodies and Apo2L/TRAIL is disappointing, recent published data shows that anti-DR5 antibodies can synergize with Apo2L/TRAIL to increase killing of cancer cells, so this approach could have increased efficacy in patients [38]. Since many patients with pancreatic cancer present with metastases, the effect of DR5 monoclonal antibody treatment on disseminated disease will also need further assessment. Previous work by Hermann et al. suggests that CSCs play a major role in the distant metastases of pancreatic cancer [39]. If true, then these therapies may help treat disseminated disease. In a preclinical PDX model, we have previously shown that liver metastases from an Apo2L/TRAIL sensitive, orthotopically-implanted patient tumor remained sensitive to Apo2L/TRAIL, suggesting that death receptor targeting agents could prove useful in advanced disease [15]. Ultimately, further investigation is still needed to determine the exact role of drozitumab in the treatment of pancreatic cancer, but our current findings reveal significant promise for its future application.

## Conclusions

Overall, our findings provide new evidence which support further development of death receptor targeting therapies. Here, we confirm that pancreatic CSCs derived from patient tumor xenografts possess increased tumorigenic potential compared with the non-stem cell bulk population. Using in vivo assays in SCID mice, we showed that small numbers of purified CSCs could initiate tumor formation; however, drozitumab treatment of mice engrafted with CSCs completely prevented tumor formation, indicating that the antibody is effective at inducing apoptosis in CSCs, as has been shown for tigatuzumab [27]. Furthermore, not only are CSCs preferentially killed by antibody-mediated activation of DR5, but our findings show for the first time that continued, long-term killing of the remaining DR5- negative cells may occur as a result of expression of newly synthesized receptors on the cell surface, based on the detection of mRNA expression. However, a more in depth investigation of the mechanisms underlying this DR5 expression is needed. Ultimately, these finding demonstrate that drozitumab has potential to be used in conjunction with other therapies to improve treatment of pancreatic cancer.

## Methods

### Cell culture and reagents

Panc-1 cells were purchased from ATCC and cultured at 37 °C with 5 % CO_2_. All culture reagents were purchased from Cellgro (Corning, NY). Panc-1 cells were maintained in RPMI-1640 supplemented with 10 % heat inactivated FBS, 1 % L-glutamine, and 1 % penicillin-streptomycin. PDXs were harvested from SCID mice, dissociated (see below), and cultured with RPMI-1640 supplemented with 20 % heat inactivated FBS, 1 % human serum (Valley Biomedical, Knoxville, TN), 1 % L-glutamine, and 1 % penicillin-streptomycin.

### Isolation of CSCs and establishment CSC derived tumors from patient tumor xenografts

De-identified pancreatic cancer specimens were acquired through the RPCI Tissue Procurement Service; patient consent was obtained for the use of “Remnant clinical biospecimans” in accordance with the Institutional Review Board at RPCI.

Tumor tissues were immediately implanted subcutaneously (SQ) into SCID mice and allowed to engraft [14]. Successfully engrafted tumors were serially passaged into other SCID mice or cryopreserved (10 % DMSO, 50 % FBS and 40 % RPMI) for future studies.

To generate cancer stem cell-derived xenografts, patient tumor xenografts were dissociated for 2 h with Type IV collagenase (Worthington, Lakewood, NJ) and strained through 70 μm filters. CSCs were labeled with anti-CD44 (BD Biosciences, Franklin Lakes, NJ), anti-CD24 (BD Biosciences, Franklin Lakes, NJ), and anti-CD326/ESA (Miltenyi, San Diego, CA), and then isolated by FACSort using a FACSAria (BD Biosciences, Franklin Lakes, NJ). Purified CSCs were re-suspended in BD Matrigel Growth Factor Reduced then injected SQ into the lower abdomen of SCID mice and monitored for growth. Cells negative for all three markers were also recovered and used as experimental controls. All experiments were conducted according to approved Roswell Park Cancer Institute IACUC and IRB protocols.

### In vivo therapeutic studies

SCID mice were implanted SQ with patient tumor xenografts as previously described [14, 15]. When tumors reached 100 mm^3^, mice were treated with 200 μg of drozitumab by intraperitoneal (IP) injection. To assess the degree of in vivo apoptosis, tumors were excised approximately 5 h after treatment with drozitumab and dissociated with a Miltenyi gentleMACs Tissue Dissociator (Miltenyi, San Diego, CA) into a single cell suspension in order to minimize cellular damage. The tumor cells were then stained with anti-CD24, anti-CD44, anti-CD326/ESA, and biotinylated anti-H2K^D^ BD Biosciences, Franklin Lakes, NJ. A PE-Cy5 conjugated streptavidin (BD Biosciences, Franklin Lakes, NJ) secondary was used to label the biotinylated anti-H2K^D^. To determine the level of apoptosis, cells were permeabilized with BD Cytoperm/Cytofix (BD Bioscience), stained intracellularly for cleaved capase-3 (BD Bioscience). Staining was assessed on an LSR II flow cytometer (BD Biosciences), and data was analyzed using FCS Express software (DE Novo).

### Death receptor expression

Patient xenografts were harvested and dissociated with Type IV collagenase in RPMI-1640 media (0.8 mg/mL) overnight at 37 °C. The cells were filtered through a 70 μM filter in order to remove large debris and then washed with RPMI. Afterwards, cells were re-suspended in 2 mL of RPMI and centrifuged in a Ficoll-Percol (GE Healthcare, Buckinghamshire, UK) gradient in order to remove further debris. The cells were washed with PBS and counted with a hemocytometer. 10^6^ cells were used for staining. Human cells were identified by exclusion using anti-mouse H2K^D^ in order to identify and exclude murine stromal cells from the analysis. Tumor initiating CSCs were identified as previously described above. Further analysis of death receptor expression was done by staining with primary antibodies for DR4, DR5, DcR1, and DcR2 (all mouse IgG; Alexis, Farmingdale, NY). Mouse IgG isotype controls (Santa Cruz Biotechnology, Dallas, TX) were compared with unstained patient tumor cells. Flow cytometric analysis was performed as previously described.

### In vitro killing assays

Patient xenografts were dissociated using type IV collagenase. Debris was removed by centrifugation with Ficoll-Percol and cells were collected from the gradient interface. Live cells were counted and 10^6^ plated into a 24 well plate. After 24 h, 10 μg/mL of drozitumab and 10 μg/mL of anti-human Fc (Jackson Immunoresearch, West Grove, PA) were added to each well for 6 h in serum-free media. After, cells were stained for cancer stem cell markers and then permeabilized with BD Cytoperm/Cytofix for intracellular flow cytometry analysis of cleaved caspase-3 (BD Biosciences, Franklin Lakes, NJ) in order to determine viability.

Assessment of cell surface DR5 on patient xenografts was performed by dissociating tumors into single cell suspensions as described above and plating in culture. Panc-1 tumor cells were also cultured as described. Cells were treated with 10 μg/mL of drozitumab and 10 μg/mL of anti-human Fc (Jackson Immunoresearch, West Grove, PA) for 10 h in serum-free media. After, cells were washed with full serum media and stained for DR5 using drozitumab and anti-human Dylight 647 (Jackson Immunoresearch, West Grove, PA) at 0, 8, 24 and 48 h post-treatment. The presence of cell surface DR5 was examined by flow cytometry.

Additionally, expression of the DR5 mRNA was also analyzed after drozitumab treatment to determine if the receptor was newly synthesized or recycled to the plasma membrane from the cytoplasm of the remaining DR5− cells. mRNA was harvested from Panc-1 cell lines at 0, 8, 24 and 48 h post-treatment using the RNeasy Mini Kit (Qiagen, Germantown, MD). cDNA was synthesized with iScript cDNA synthesis kit (Bio-Rad, Hercules, CA). 250 ng of cDNA per well in a SYBR Select Master Mix (Thermofisher, Waltham, MA) was used for qPCR in a DNA Engine Peltier thermal cycler (Bio-Rad, Herculues, CA). Primers for qPCR reactions were designed as follows, DR5 Fwd 5′-GGGCCA CAGGGACACCTT-3′; DR5 Rev 5′-GCATCTCGCCCGGTTTT-3′; GAPDH Fwd 5′-CGGAGTCAACGGATTTGGTCGTAT-3′; GAPDH Rev 5′-AGCCTTCTCCATGGTGGTGAAGAC-3′. Fold changes in DR5 mRNA expression were determined by calculating the ΔCt using GAPDH as the house keeper.

## Additional files

Additional file 1:
**Figure S1.** Treatment three times per week of 12424 did not inhibit tumor growth. 12424 tumors implanted SQ into mice and given 200 μg of drozitumab 3×/week. *n* = 8 mice/group. **Figure S2.** Tumors derived from purified CSCs recapitulate the morphology of the original patient xenograft. H&E images of the patient pancreatic adenocarcinoma xenograft 14244(A) and the tumor derived from CSCs isolated from that tumor (B). **Figure S3.** In vitro treatment of tumor cells with drozitumab depletes cancer stem cells. **A**) Panc-1 pancreatic tumor cells or dissociated tumor cells from **B**) 12424 were treated in vitro with 10 μg/ml of drozitumab and 10 μg/ml of anti-human IgG Fc antibody for 12 h to ensure maximum killing. The percentages of CSCs were assessed by flow cytometry using markers for ESA, CD44, and CD24. (PPT 664 kb)

## Abbreviations

ATTC: American type culture collection
cDNA: complementary deoxyribonucleic acid
CSCs: cancer stem cells
DcR1: decoy receptor 1
DcR2: decoy receptor 2
DR4: death receptor 4
DR5: death receptor 5
FACS: fluorescence-activated cell sorting
FBS: fetal bovine serum
IACUC: Institutional Animal Care and Use Committee
IRB: Institutional Review Board
mRNA: messenger ribonucleic acid
PDX: patient tumor xenograft
qPCR: quantitative polymerase chain reaction
RPMI: Roswell Park Memorial Institute
SCID: severe combined immunodeficiency
SQ: subcutaneously
TNF: tumor necrosis factor
TRAIL: tumor necrosis factor apoptosis inducing ligand
