# Supplementary material for: Pancreatic cancer stem cells in patient pancreatic xenografts are sensitive to drozitumab, an agonistic antibody against DR5
Source: J Immunother Cancer. 2016 Jun 21;4:33. doi: 10.1186/s40425-016-0136-y (PMC4915140; doi:10.1186/s40425-016-0136-y)
Supplement: Additional file 1: — Figure S1. Treatment three times per week of 12424 did not inhibit tumor growth. 12424 tumors implanted SQ into mice and given 200 μg of drozitumab 3×/week. n = 8 mice/group. Figure S2. Tumors derived from purified CSCs recapitulate the morphology of the original patient xenograft. H&E images of the patient pancreatic adenocarcinoma xenograft 14244(A) and the tumor derived from CSCs isolated from that tumor (B). Figure S3. In vitro treatment of tumor cells with drozitumab depletes cancer stem cells. A) Panc-1 pancreatic tumor cells or dissociated tumor cells from B) 12424 were treated in vitro with 10 μg/ml of drozitumab and 10 μg/ml of anti-human IgG Fc antibody for 12 h to ensure maximum killing. The percentages of CSCs were assessed by flow cytometry using markers for ESA, CD44, and CD24. (PPT 664 kb) [file 40425_2016_136_MOESM1_ESM.ppt]

## Slide 1
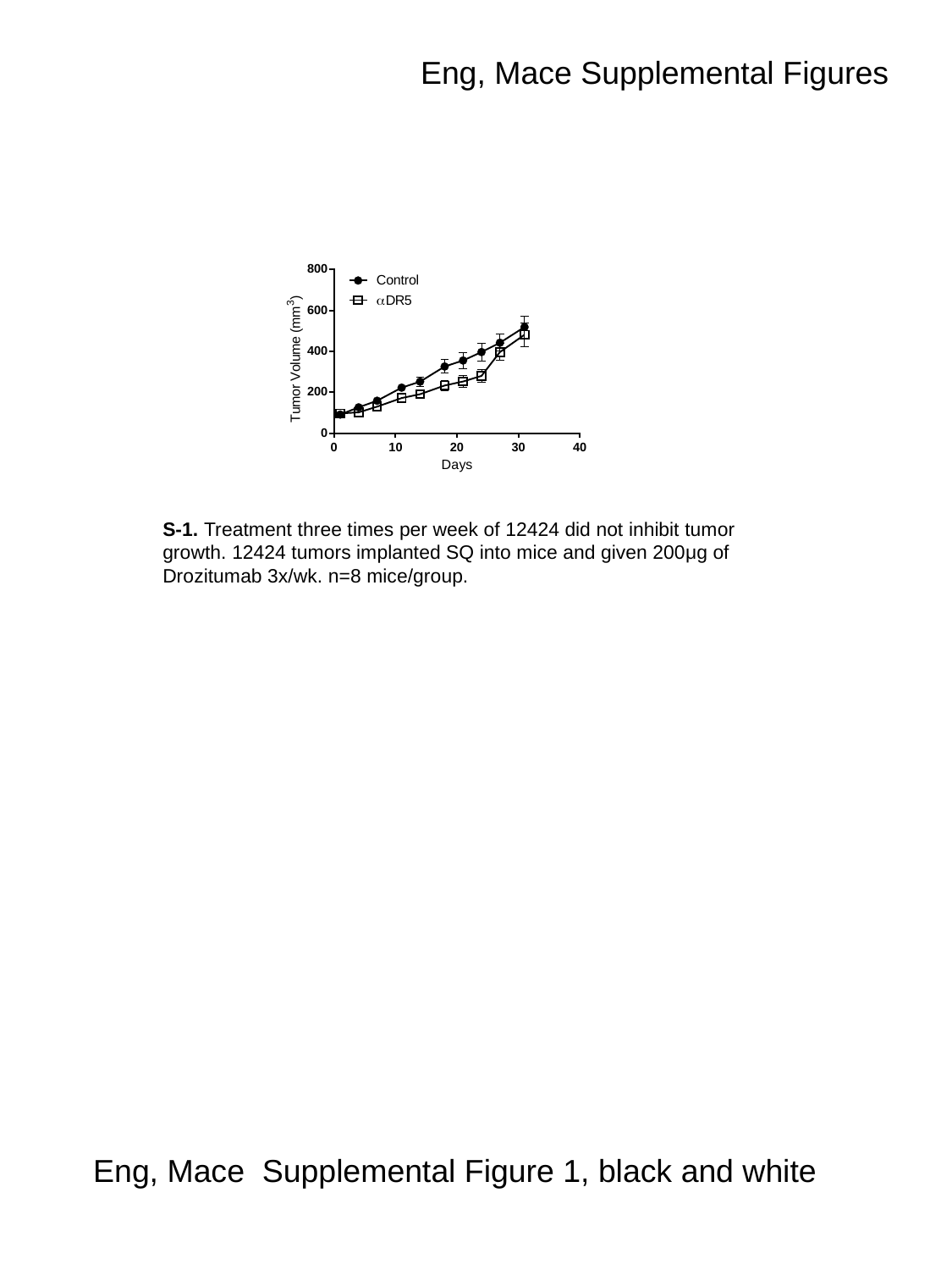

Eng, Mace Supplemental Figures
S-1. Treatment three times per week of 12424 did not inhibit tumor growth. 12424 tumors implanted SQ into mice and given 200μg of Drozitumab 3x/wk. n=8 mice/group.
Eng, Mace Supplemental Figure 1, black and white

## Slide 2
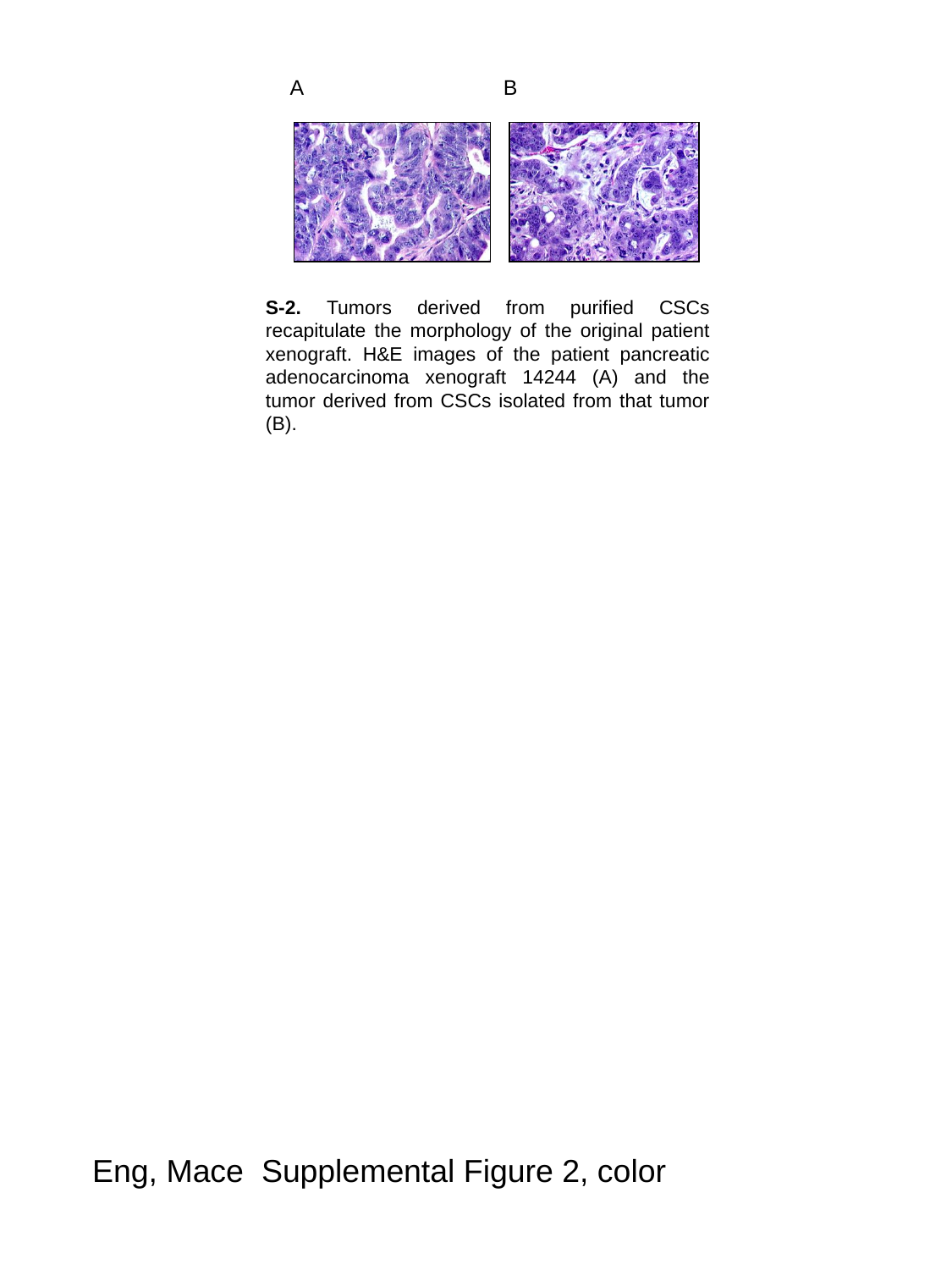

A
B
S-2. Tumors derived from purified CSCs recapitulate the morphology of the original patient xenograft. H&E images of the patient pancreatic adenocarcinoma xenograft 14244 (A) and the tumor derived from CSCs isolated from that tumor (B).
Eng, Mace Supplemental Figure 2, color

## Slide 3
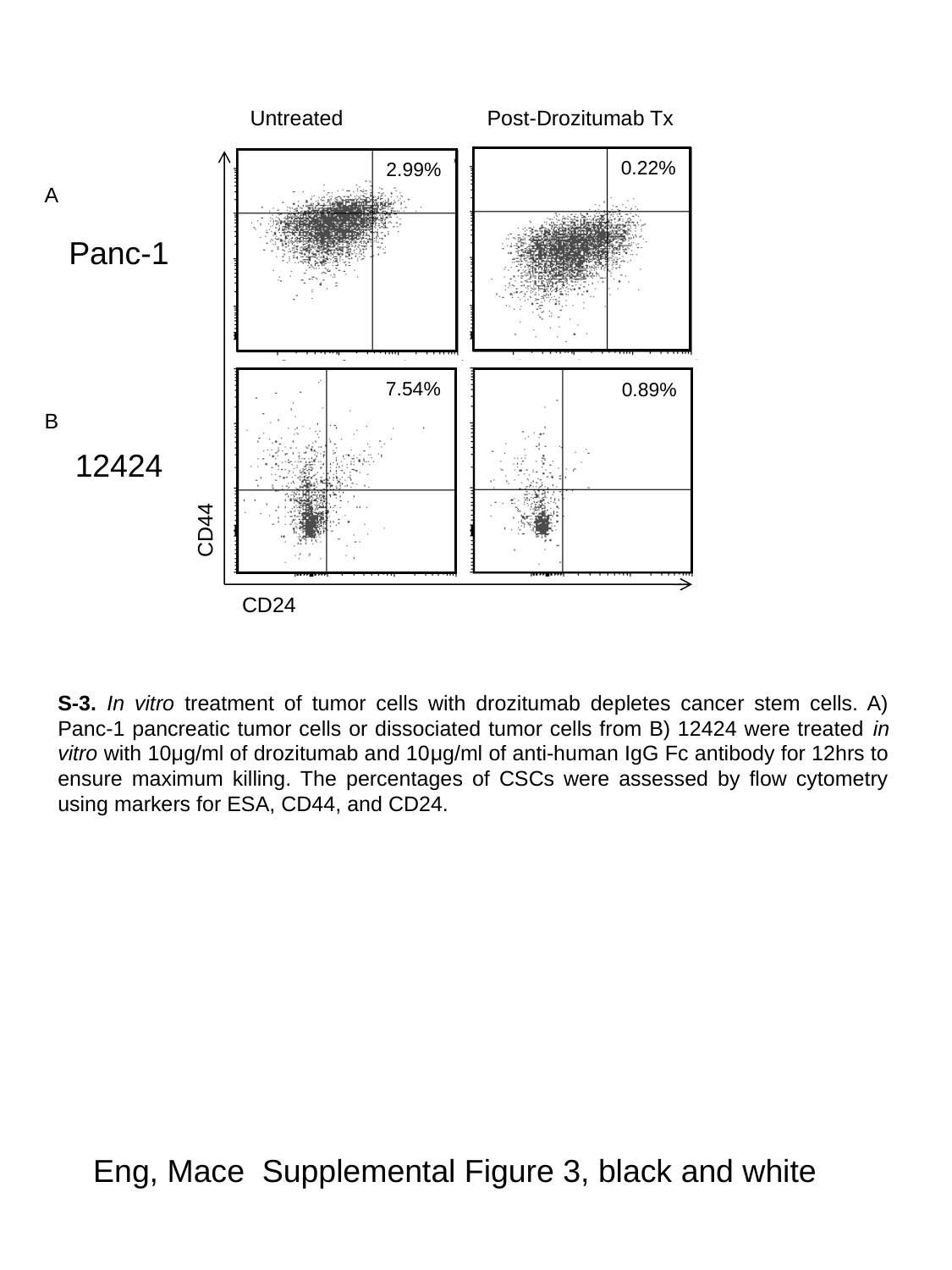

Untreated
Post-Drozitumab Tx
0.22%
2.99%
A
Panc-1
7.54%
0.89%
B
12424
CD44
CD24
S-3. In vitro treatment of tumor cells with drozitumab depletes cancer stem cells. A) Panc-1 pancreatic tumor cells or dissociated tumor cells from B) 12424 were treated in vitro with 10μg/ml of drozitumab and 10μg/ml of anti-human IgG Fc antibody for 12hrs to ensure maximum killing. The percentages of CSCs were assessed by flow cytometry using markers for ESA, CD44, and CD24.
Eng, Mace Supplemental Figure 3, black and white
